# Role of the Posterior Fat Pad Sign in Treating Displaced Extension Type Supracondylar Fractures of the Pediatric Elbow Using the Blount Method

**DOI:** 10.7759/cureus.6024

**Published:** 2019-10-29

**Authors:** Panagiotis V Samelis, Eftychios Papagrigorakis, Sotiris Ellinas

**Affiliations:** 1 Orthopaedics, Children’s General Hospital Panagiotis & Aglaia Kyriakou, Athens, GRC; 2 Orthopaedics, KAT Trauma Hospital, Athens, GRC; 3 Orthopaedics, General Children’s Hospital Panagiotis & Aglaia Kyriakou, Athens, GRC

**Keywords:** elbow, paediatric, supracondylar, fracture, gartland, blount, fat, pad

## Abstract

The posterior fat pad sign (PFPS) on the lateral elbow X-ray is useful in the diagnosis of a suspected nondisplaced fracture about the elbow after a subtle injury. However, the presence of a PFPS hallmarks a continuous posterior periosteum-posterior capsule of the distal humerus. This anatomic structure is crucial for the stable anatomic reduction of a displaced extension type supracondylar fracture. Subsequently, the presence of a PFPS may predict the successful nonoperative treatment of a Gartland III-type fracture by means of the Blount method, implying less implant-related morbidity and less intraoperative radiation exposure for the patient and surgeon. Furthermore, it is concluded that a PFPS-positive displaced extension type supracondylar fracture is definitively classified as a Gartland III and not a Gartland IV-type fracture. A retrospective study of 75 displaced extension type supracondylar elbow fractures was performed. All fractures had an initial attempt at a closed reduction under general anesthesia. A stable reduction in 120 degrees of elbow flexion without vascular compromise of the affected limb was achieved in 45 fractures, which were splinted in this position. In the remaining 30 cases, fracture reduction was either inadequate or was achieved with excess elbow flexion, which impaired distal blood flow. These fractures were pinned percutaneously and splinted in 90 degrees of elbow flexion. The two treatment groups were retrospectively compared for the presence of a PFPS. The displaced extension type supracondylar fractures, which were treated successfully by the Blount method, had a statistically significant higher prevalence (chi-square-Yates =4.91, p<0.05) of a positive PFPS (28/45 patients, 62.22%) compared to the fractures treated by closed reduction and percutaneous pinning (10/30, 33.33%). No vascular complications were observed. The long-term outcome did not differ between groups.

## Introduction

Supracondylar fractures of the humerus represent about two-thirds of elbow fractures in children. The non-dominant arm of boys aged five to seven years is most frequently involved. Extension type supracondylar fractures are the most frequent (99%) fracture type [1]. The mechanism of injury is secondary to a fall onto an outstretched hand [2-3]. The remaining fractures are classified as flexion type supracondylar fractures and are the result of a direct blow to the flexed elbow [2-3]. Extension type supracondylar fractures are further classified into three types, according to the Gartland classification [4]. Gartland I-type fractures are minimally displaced fractures while Gartland II-type fractures are complete fractures of the anterior cortex with a continuous posterior cortex. Gartland III-type fractures are completely displaced fractures, without contact of both the anterior and posterior cortices of the distal humeral metaphysis. Provided that adequate reduction has been obtained, Gartland I and II-type fractures are usually stabilized by nonsurgical means while Gartland III fractures are almost routinely treated by closed reduction and percutaneous pinning (CRPP) [2-4]. In 20% of the cases, open reduction may be necessary to obtain an acceptable result [1]. However, it seems that the displaced extension type supracondylar fractures of the pediatric elbow, collectively described as Gartland III-type fractures, are not a homogenous group. Depending on fracture stability during reduction maneuvers under anaesthesia, a new fracture type - the Gartland IV-type fracture - has been recently described: Gartland III-type fractures are unstable in extension while Gartland IV-type fractures are unstable in both flexion and extension, due to the complete rupture of the posterior capsule-posterior periosteum of the distal humerus [5].

Decades ago, Blount described a method of nonsurgical stabilization of the extension type supracondylar fractures of the elbow by means of elbow hyperflexion [6]. However, this method implies an increased risk of neurovascular complications and compartment syndrome secondary to rising intracompartmental pressures of the forearm when the elbow is flexed beyond 90 degrees [7]. On the other hand, neither CRPP nor the Blount method seems to differ in terms of predicting a better outcome of the treatment of displaced extension type supracondylar fractures of the elbow [8].

Recent studies support the Blount method for the treatment of Gartland II and III-type fractures provided that a stable anatomic reduction of the fracture without vascular compromise of the limb is achieved [9-12]. The aim of this study is to investigate whether the presence of a posterior fat pad sign (PFPS), that is frequently obvious on the first lateral elbow projection, i.e. the elbow X-ray obtained on patient admission, indicates a Gartland III-type supracondylar fracture that is inherently stable in flexion according to the four-type Gartland classification [5]. This fracture might benefit from conservative treatment by means of the Blount method.

## Materials and methods

**Figure 1:**
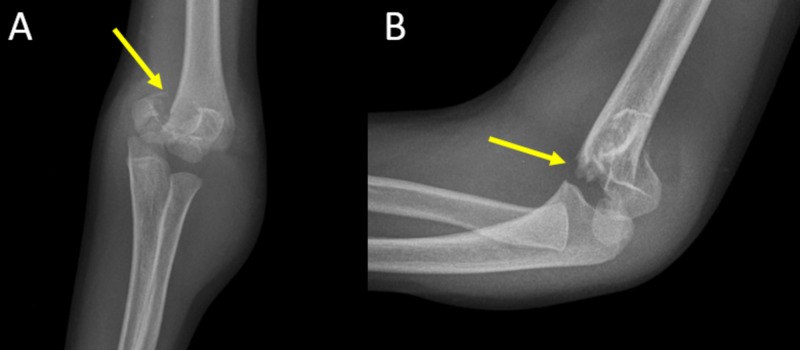
Anteroposterior (A) and lateral (B) x-ray view of the elbow of a five-year-old boy with a displaced extension type supracondylar fracture Arrows indicate the location of the fracture.

**Figure 2:**
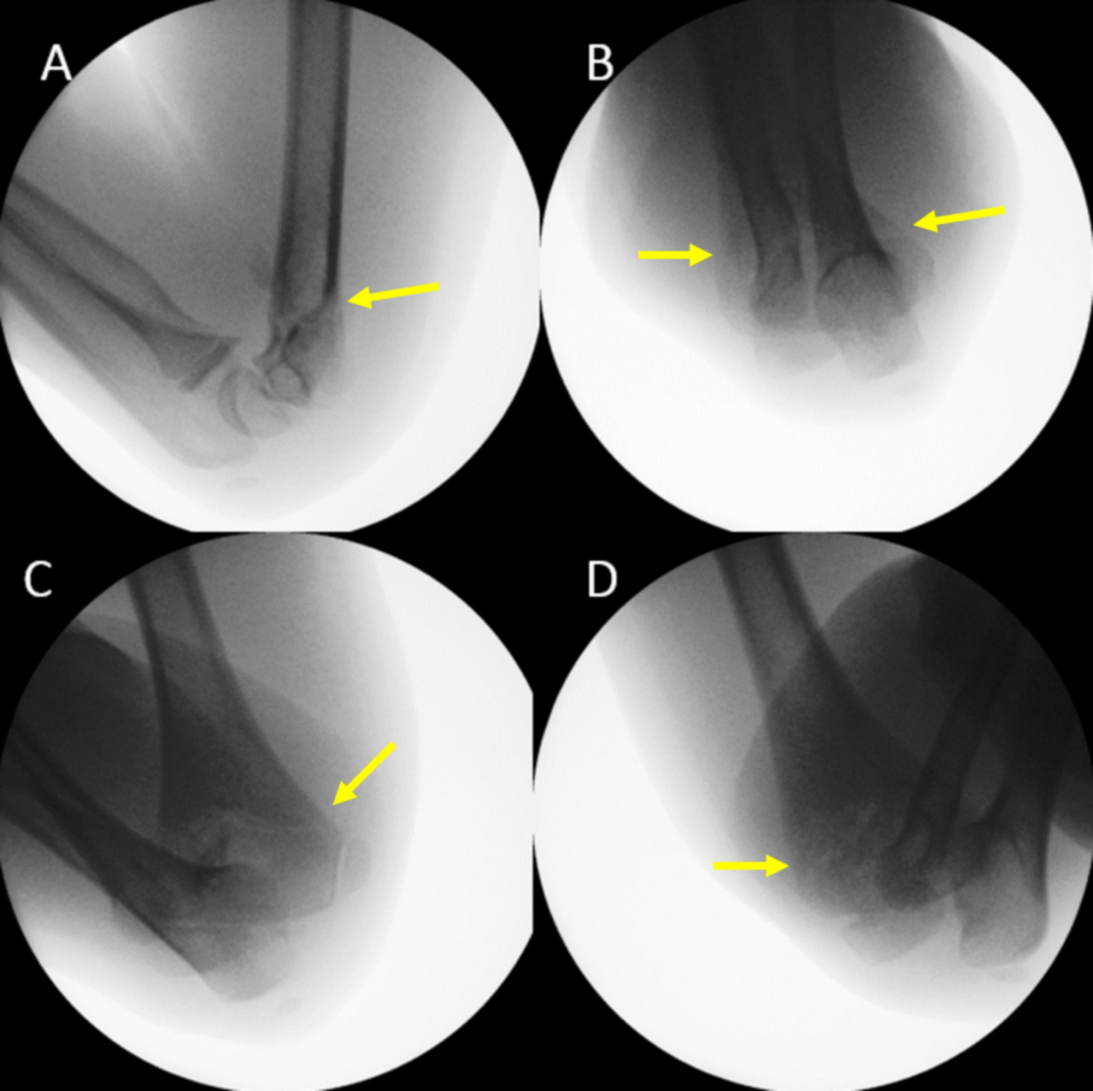
Radiographic controll of fracture reduction under general anesthesia Lateral view (A), Jones view (B), external view (C), internal oblique view (D) of the elbow. Only a continuous diaphyseal-metaphyseal contour of the humerus on all projections is accepted. Arrows indicate the location of the fracture after reduction.

**Figure 3:**
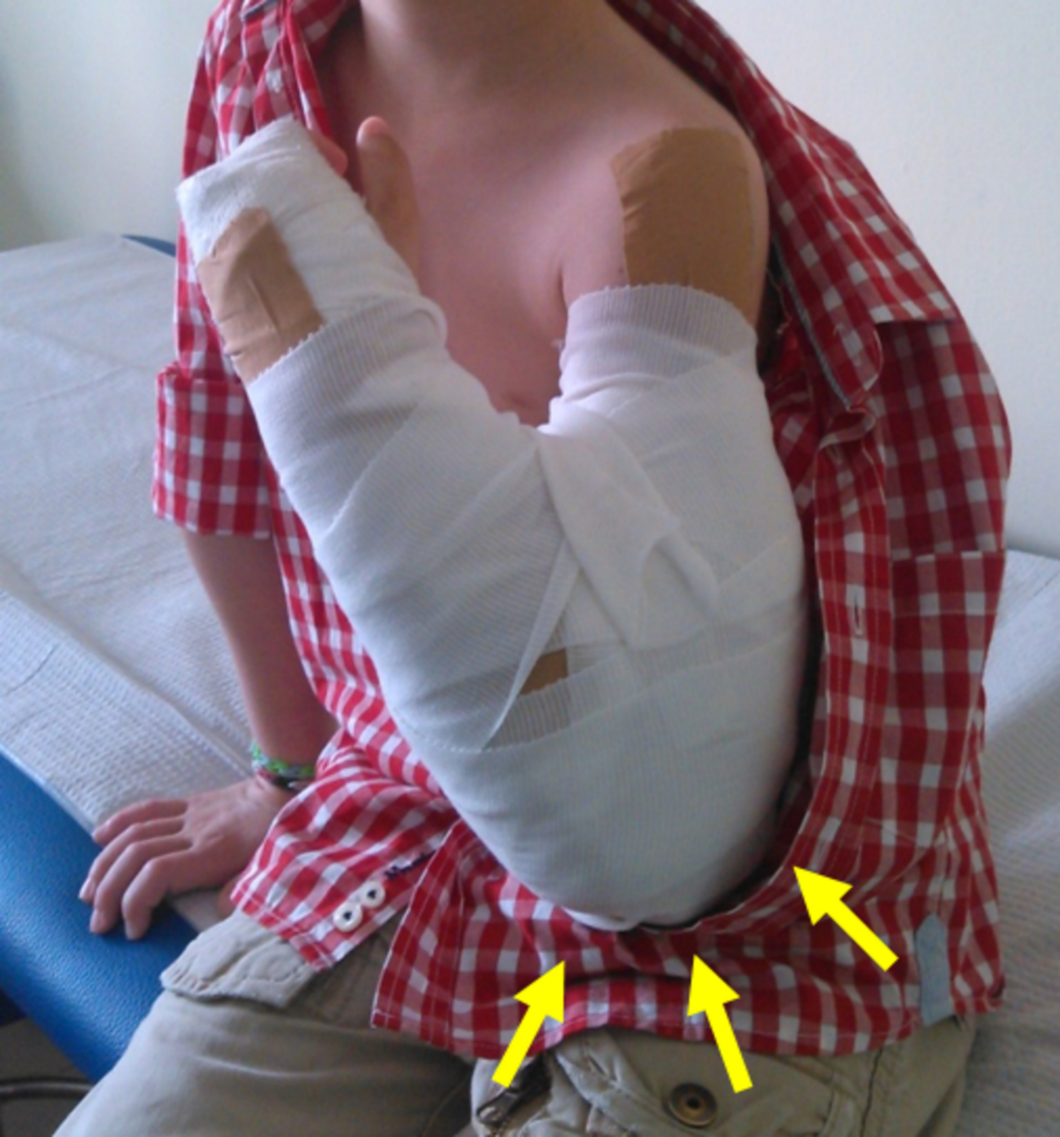
Patient in Figure 1: Fracture reduction is maintained in a long arm cast with the elbow flexed (Blount method) Arrows indicate the stabilization of the fracture with elbow flexion at 120 degrees.

A retrospective study of 75 children, 49 boys and 26 girls, aged 4.3 to 8.6 years, diagnosed with a displaced extension type supracondylar elbow fracture, treated between 2011 and 2018, was performed (Figure 1). Only fractures with a palpable radial pulse and normal nerve function distal to the injury level were included. All fractures had an initial attempt of conservative treatment by means of the Blount method: closed reduction under anesthesia and stabilization in 120 degrees of elbow flexion. Patients were divided into two groups: The first group (Group A) consisted of 45 fractures that were treated successfully by means of the Blount method. The anatomic reduction was confirmed with additional X-ray projections, including the Jones projection and oblique projections of the elbow as well (Figure 2). A long arm cast with the elbow flexed was placed in order to secure fracture reduction (Figure 3). The second group (Group B) included the remaining 30 fractures, in which the Blount method failed to provide stable anatomic fracture reduction. These fractures underwent CRPP and were splinted in 90 degrees of elbow flexion. The patients were observed clinically and radiologically on a weekly basis. Splints and pins were removed after three to four weeks, depending on the radiologically confirmed fracture healing.

**Figure 4:**
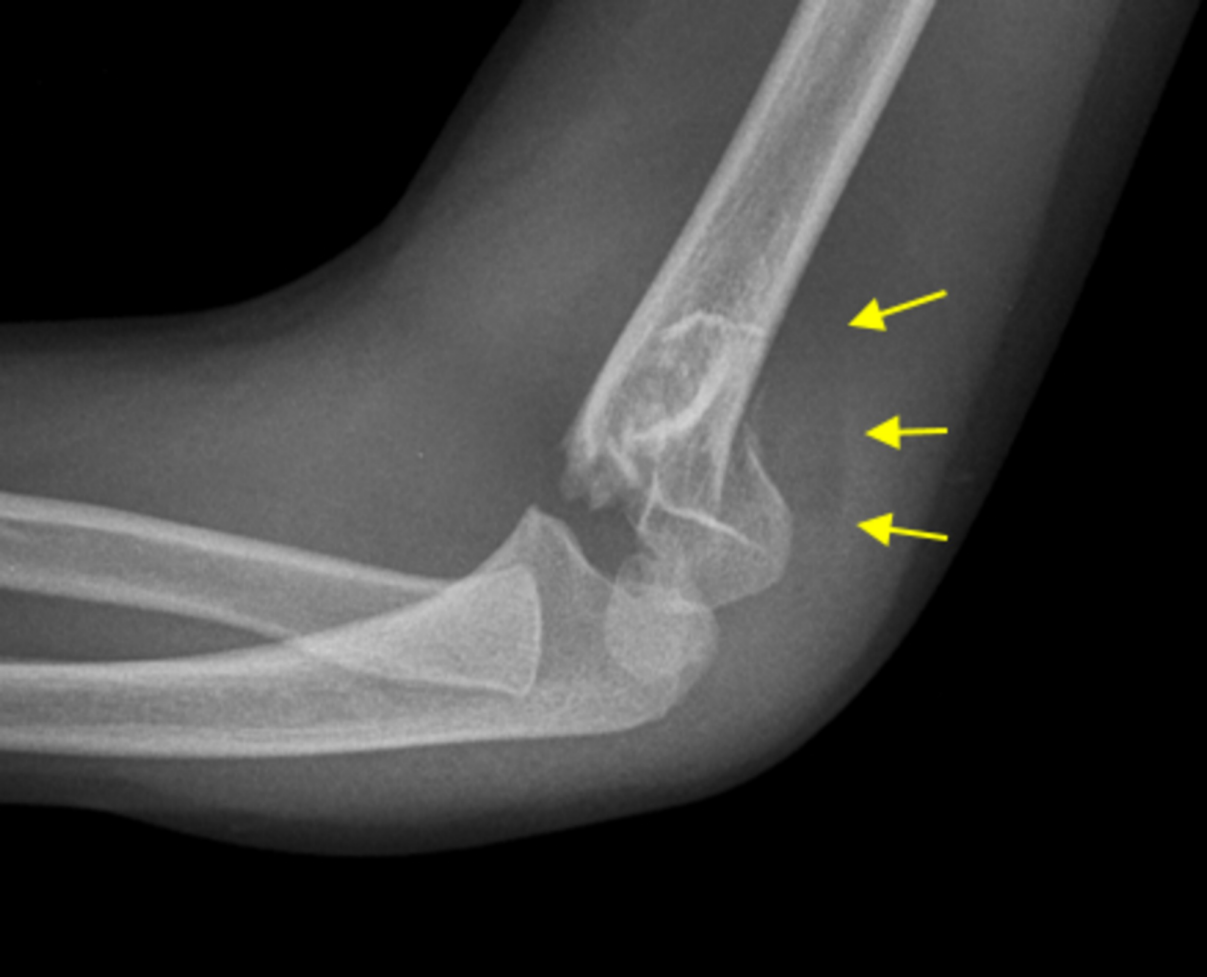
Lateral X-ray elbow projection of the patient in Figure 1 Arrows indicate a posterior fat pad sign.

The two groups were retrospectively compared for the presence of a PFPS on the lateral X-ray of the injured elbow (Figure 4). Chi-square, corrected after Yates, at a statistical significance level of 0.05, was the selected statistical method.

## Results

The long-term outcomes and complication rates were similar between groups. All patients had a functional elbow range of motion within six to eight weeks post-injury. Full range of motion was achieved within six to nine months. One case of cubitus varus was observed in each group. One radial nerve and one anterior interosseous nerve apraxia, which resolved spontaneously within three months, were observed in the Blount group and the CRPP group, respectively. No vascular complications or compartment syndromes were observed in either group.

The two groups differed significantly (Chi square=4.91, correction after Yates, p<0.05) when retrospectively compared for the presence of a PFPS: 28/45 (62.22%) of the fractures that were treated successfully by the Blount method presented a PFPS on the lateral elbow X-ray (PFPS-positive). On the other hand, 20/30 (66.6%) of the fractures that failed conservative treatment and were finally pinned did not present a PFPS (PFPS-negative). The likelihood of successful treatment by means of the Blount method is higher in PFPS-positive as compared to PFPS-negative fractures (odds ratio=3.29) (Table 1).

**Table 1:** Children with displaced extension type supracondylar fractures treated between 2011-2018 Group A includes the fractures that were successfully treated by means of the Blount method. Group B includes the fractures that failed a stable anatomic reduction with the Blount method and subsequently underwent CRPP. CRPP: closed reduction percutaneous pinning, PFPS: posterior fat pad sign

	Group A: successful Blount method	Group B: CRPP	
PFPS-positive fractures	28	10	38
PFPS-negative fractures	17	20	37
	45	30	n=75
% of PFPS-positive fractures in each group	62.22	33.33	

## Discussion

It has been questioned if surgeons will find the four-type Gartland classification useful [2]. Practically, the addition of the Gartland IV-type fracture did not alter the currently recommended method of treatment of the originally described Gartland III-type fractures by means of CRPP. CRPP remains, for most surgeons, the standard treatment for displaced extension type supracondylar fractures of the elbow, whether these fractures are of the Gartland III type or the Gartland IV type [13]. Furthermore, the distinction between the Gartland III and Gartland IV-type fractures is a strict intraoperative finding and cannot be concluded on patient admission. On the other hand, the preoperative radiologic diagnosis of a Gartland IV-type fracture is still uncertain [14].

CRPP bears some problems per se: implant-related morbidity, such as pin migration, infection, or iatrogenic nerve injury, are potential complications of CRPP. Furthermore, repeat attempts to achieve anatomic reduction and proper pin placement may unnecessarily extend the duration of the surgical procedure and increase the radiation exposure of the patient and the surgeon. Blount described a method of conservative treatment of displaced extension type supracondylar fractures of the elbow that is based on the stabilizing effect of a continuous posterior periosteum and the triceps tendon [9]. These anatomic structures represent a soft tissue constraint that favors the stable anatomic reduction of a displaced extension type supracondylar fracture of the elbow.

The question is whether there is a clue that indicates posterior soft tissue integrity. This finding, detected not intraoperatively but on patient admission and initial examination, might help in treatment selection for these fractures: CRPP or the Blount method.

First described by Norel decades ago, but still valid, the only utility of a posttraumatic PFPS was to suspect an occult, nondisplaced fracture about the elbow joint after subtle injury [15-17]. However, this sign has one additional interpretation. The presence of a PFPS indicates hematoma formation at the fracture site that is contained by a continuous posterior capsule-posterior periosteum complex. The latter is an important soft tissue constraint that promotes the anatomic reduction of the supracondylar fracture. Consequently, the presence of the PFPS implies posterior soft tissue integrity that is a prerequisite for the successful treatment of displaced extension type supracondylar fractures by means of the Blount method [9].

**Figure 5:**
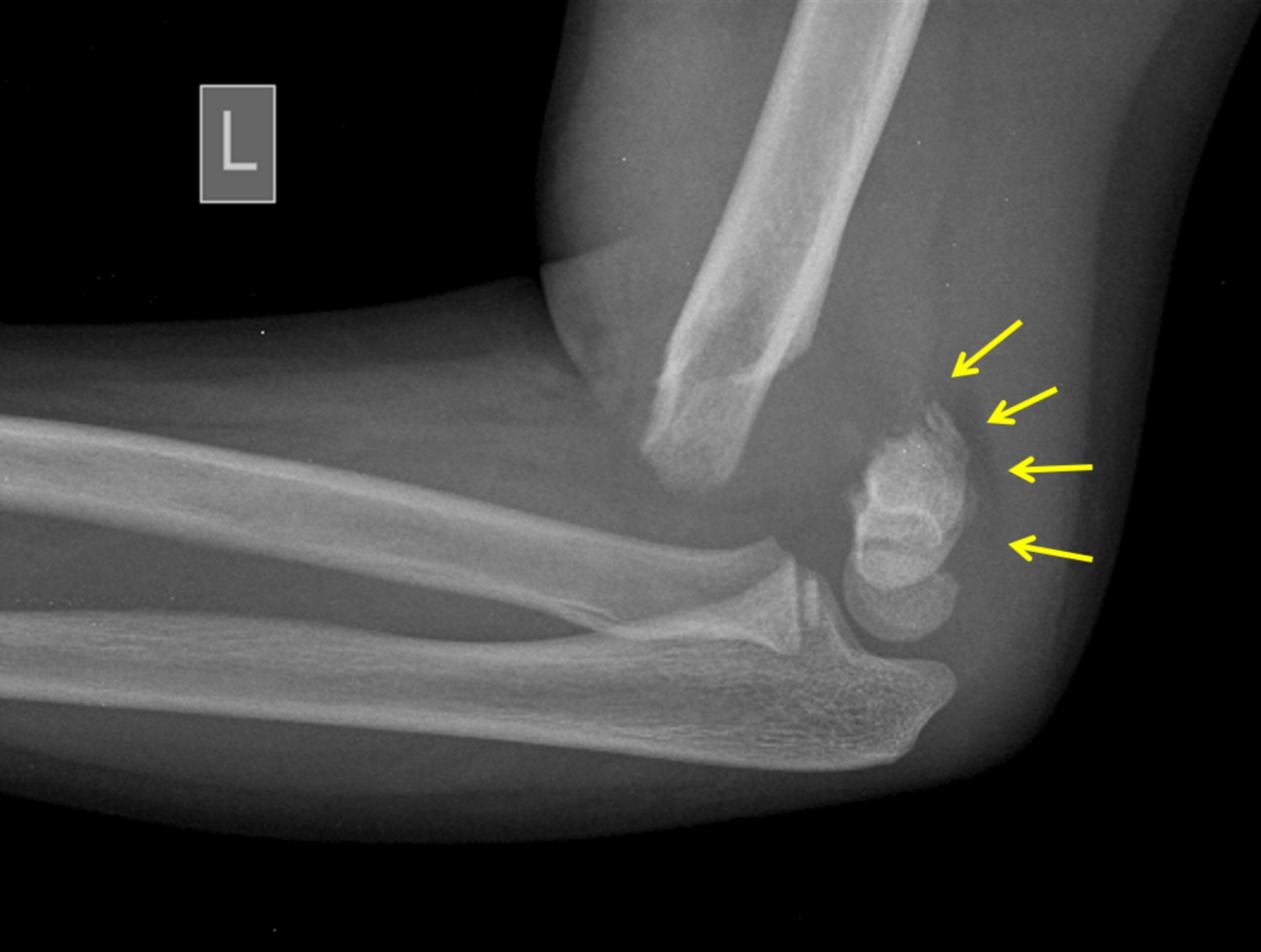
A seven-year-old boy with a PFPS-positive displaced extension type supracondylar fracture of the left elbow Arrows indicate a posterior fat pad sign due to a continuous periosteum-posterior elbow capsule complex. PFPS: posterior fat pad sign

**Figure 6:**
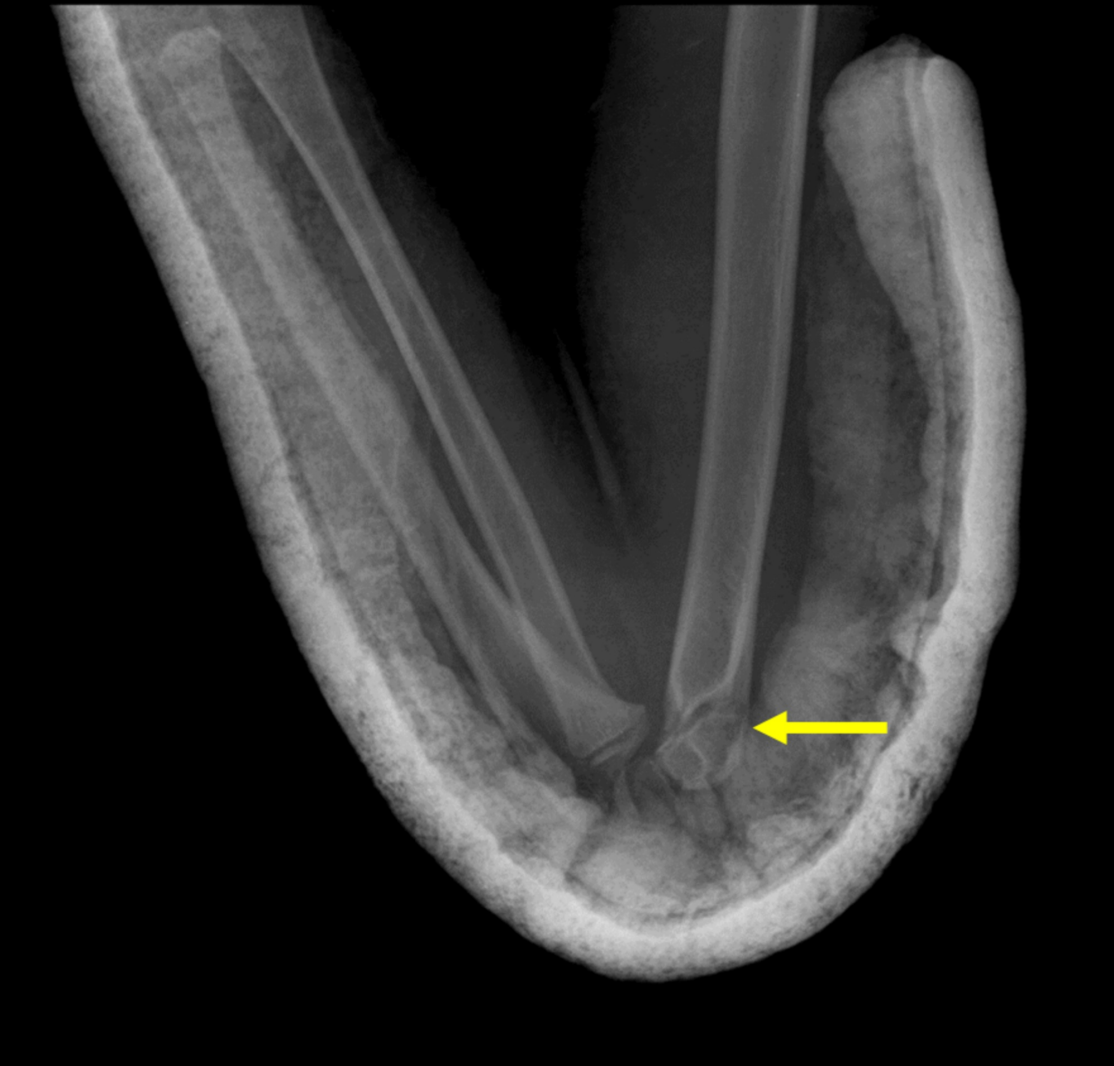
Patient in Figure 5: anatomic reduction of a PFPS-positive displaced extension type supracondylar fracture of the elbow by means of the Blount method. Arrow indicates the location of the fracture after reduction. PFPS: posterior fat pad sign

According to this study, the PFPS is a simple radiographic sign to distinguish Gartland III and Gartland IV-type fractures: PFPS-positive fractures are safely classified as Gartland III-type fractures (Figure 5). True Gartland III-type fractures are stable in flexion and may benefit from nonoperative treatment with elbow hyperflexion (Figure 6). However, CRPP is mandatory in Gartland IV-type fractures. Fracture comminution or obliquity may explain the failure of conservative treatment in PFPS-positive fractures (10/30, 33.3%) [18].

This study has some limitations. Many patients were referred to the hospital without a clear history of previous manipulations to reduce the fracture by the physician, who first examined the patient. Manipulations to reduce the fracture could potentially rupture the posterior capsule - posterior periosteum complex, thus leading to drainage of the fracture hematoma to the surrounding tissues and subsequently to the loss of a PFPS. Delay of patient admission (hours, next day) prior to the radiologic examination may also result in the loss of a PFPS, even in the presence of an intact posterior periosteum-posterior capsule complex due to gradual drainage of the fracture hematoma. These factors explain the increased number of PFPS-negative displaced extension type supracondylar fractures that were treated successfully with the Blount method (17/45, 37.78%).

## Conclusions

The detection of a posterior fat pad sign on the lateral X-ray of the pediatric elbow with a displaced extension type supracondylar fracture indicates a continuous posterior capsule-posterior humeral periosteum complex. The integrity of this anatomic structure is, by definition, a prerequisite for successful treatment by means of the Blount method. Elbow flexion increases tension on the posterior capsule-posterior periosteum complex and stabilizes the fracture in the reduced position. Subsequently, PFPS-positive displaced extension type supracondylar fractures of the elbow are safely classified as Gartland III and not as Gartland IV-type fractures. PFPS-positive displaced extension type supracondylar fractures of the elbow are stable in flexion and may benefit from conservative treatment, provided that the fracture is not oblique or comminuted and the neurovascular status distal to the fracture level is normal.
